# Co-morbidity and visual acuity are risk factors for health-related quality of life decline: five-month follow-up EQ-5D data of visually impaired older patients

**DOI:** 10.1186/1477-7525-7-18

**Published:** 2009-02-25

**Authors:** Ruth MA van Nispen, Michiel R de Boer, Janneke GJ Hoeijmakers, Peter J Ringens, Ger HMB van Rens

**Affiliations:** 1 VU University Medical Center, Department of ophthalmology, PO Box 7057, 1007 MB Amsterdam, the Netherlands; 2 Institute for Research in Extramural Medicine, VU University Medical Center, Amsterdam, the Netherlands; 3 Institute of Health Sciences, VU University, Amsterdam, the Netherlands; 4 Elkerliek Hospital, Department of ophthalmology, Helmond, the Netherlands

## Abstract

**Background:**

Co-morbidity is a common phenomenon in the elderly and is considered to be a major threat to quality of life (QOL). Knowledge of co-existing conditions or patient characteristics that lead to an increased QOL decline is important for individual care, and for public health purposes. In visually impaired older adults, it remains unclear which co-existing conditions or other characteristics influence their health-related QOL. Our aim was to present a risk profile of characteristics and conditions which predict deterioration of QOL in visually impaired older patients.

**Methods:**

Analyses were performed on data from an observational study among 296 visually impaired older patients from four Dutch hospitals. QOL was measured with the EuroQol-5D (EQ-5D) at baseline and at five-month follow-up. Nine co-existing condition categories (musculoskeletal; diabetes; heart; hypertension; chronic obstructive pulmonary disease (COPD) or asthma; hearing impairment; stroke; cancer; gastrointestinal conditions) and six patient characteristics (age; gender; visual acuity; social status; independent living; rehabilitation type) were tested in a linear regression model to determine the risk profile. The model was corrected for baseline EQ-5D scores. In addition, baseline EQ-5D scores were compared with reference scores from a younger visually impaired population and from elderly in the general population.

**Results:**

From the 296 patients, 50 (16.9%) were lost to follow-up. Patients who reported diabetes, COPD or asthma, consequences of stroke, musculoskeletal conditions, cancer, gastrointestinal conditions or higher logMAR Visual Acuity values, experienced a lower QOL. After five months, visual acuity, musculoskeletal conditions, COPD/asthma and stroke predicted a decline in QOL (R^2 ^= 0.20). At baseline, the visually impaired older patients more often reported moderate or severe problems on most EQ-5D dimensions than the two reference groups.

**Conclusion:**

In visually impaired older patients, visual acuity, musculoskeletal conditions, COPD/asthma and stroke predicted a relatively rapid decline in health-related QOL. With this risk profile, a specific referral by the ophthalmologist to another sub-specialty may have a beneficial effect on the patient's health-related QOL. A referral by the ophthalmologist or optometrist to a multidisciplinary rehabilitation service seems appropriate for some patients with co-morbidity. The current results need to be confirmed in studies using pre-structured questionnaires to assess co-morbidity.

## Background

The co-occurrence of chronic conditions is a common phenomenon in the elderly and is considered to be a major threat to quality of life (QOL). Several studies report an association between the number of conditions and QOL, where a higher number of diseases is related to deterioration of physical functioning [1-4], or social and psychological functioning [5]. The prevalence rates of several conditions, including having several chronic conditions at once, increase with age [6].

The same applies to older adults with a visual impairment or blindness. Large population-based studies in the more developed countries indicate a prevalence of visual impairment and blindness ranging from 0.6–2.1% and 0.1–0.9%, respectively [7]. However, Klaver et al., who compared data from large prevalence studies in developed countries, showed that the prevalence of visual impairment and blindness increased rapidly after about 70 years of age [8]. In their study, the most common causes of visual impairment and blindness were age-related cataract and age-related macular degeneration (AMD). Due to demographic aging, these numbers are expected to increase and this group of patients will cause an increased demand for ophthalmic consultations [9]. Moreover, studies among visually impaired older patients found that co-morbidity was often reported. For example, Brody et al. found that 78% of older patients reported to have at least one other condition in addition to AMD. In our own patient population of visually-impaired older adults with a variety of eye conditions, 75% reported to have other conditions in addition to their eye disease [10]. Langelaan et al. reported that different chronic conditions have a different impact on health-related QOL [11]. Moreover, the combination of certain conditions may cause an additive or synergistic effect on QOL [1,12]. Insight into those combinations that lead to an increased QOL decline is important for the individual care of patients, and for public health purposes [12]. For older patients with an eye condition it is not yet known which co-existing conditions lead to an increased vulnerability in terms of health-related QOL or a decline in QOL.

In addition to co-existing conditions, it is expected that other characteristics of visually impaired patients (e.g. visual acuity and socio-demographics) may also influence their health-related QOL. Another consideration is that because ophthalmologists (like other sub-specialties) have limited time per patient they mainly concentrate on the eyes and less on the broader aspects of health. Assuming that knowledge of specific factors can further assist ophthalmologists in the care of their patients, the present study aims to create a risk profile of patient characteristics and self-reported co-existing conditions which predict a relatively rapid deterioration in health-related QOL.

## Methods

### Design

Secondary analyses were performed on data from a non-randomized follow-up study, which was initially set-up to investigate the longitudinal effect in terms of vision-related QOL of optometric and regional multidisciplinary rehabilitation services [13-15].

### Patients

Consecutive patients were recruited from the ophthalmology departments of one university hospital and three general hospitals in the Netherlands between July 2000 and January 2003. The eligibility requirements for inclusion in the non-randomized study were referral to low-vision services for the first time by an ophthalmologist, age over 50 years, no previous contact with low-vision rehabilitation services, irreversible vision loss, adequate understanding of the Dutch language and adequate cognitive abilities, which were assessed in communication with the ophthalmologist. Patients who met the inclusion criteria were informed about the study and were invited to participate. Written consent was obtained from all participants, which included permission for us to use their self-administered questionnaire data. The study protocol was approved by the Medical Ethics Committee of the VU University Medical Center Amsterdam, and was conducted according to the principles of the Declaration of Helsinki.

### Measurements

#### Health-related QOL

QOL was assessed at baseline and at five-month follow-up with part of the translated EuroQol instrument. The EuroQol is considered to be a generic measure of health status [16] and consists of the EuroQol 5-Dimensions (EQ-5D) and the EuroQol Visual Analogue Scale (EQ-VAS). The EQ-5D consists of five questions covering the dimensions 'mobility' (walking about, confined to bed), 'self-care' (washing oneself or getting dressed), 'usual activities' (work, study, household, family or leisure), 'pain or discomfort' and 'anxiety or depression'. Each dimension has three levels to describe the severity of problems, namely: 1) no problems, 2) moderate problems, and 3) severe problems. In a descriptive system, a respondent's health state is then defined by combining the three levels of severity on each of the five dimensions, which allows for a possible 243 (= 3^5^) health states to be defined, e.g. 11111, 12322, 22123, etc. Furthermore, for every individual a single health state value, or utility, can be calculated. These health state values are set on a scale ranging from 0 (which corresponds to death) to 1 (which corresponds to a state of perfect health). Negative values correspond to a state 'worse than death'. Moreover, valuations of the health states have been made available for the Dutch general public [17]; these health state values are referred to as the EQ-5D_index_. In the present study we did not use the EQ-VAS but chose to use the EQ-5D_index _as the main outcome measure in the prediction models because it encompasses the separate dimensions of QOL in which we were particularly interested. We used the official Dutch translation of the EQ-5D [18] and reported the most common descriptive health states for our study population. The EQ-5D has been extensively validated, also for Dutch healthy individuals [19]. There is extensive documentation  on its construct validity, reliability, and responsiveness for both general and disease-specific populations.

#### Prognostic factors

First, at baseline and at five-month follow-up patients were asked by means of an open-ended question to report whether they suffered from any condition other than their eye disease. Afterwards, the self-reported ailments and complaints were organized into 13 condition categories [20]: 1) musculoskeletal disorders (e.g. arthritis, rheumatic disease, chronic back problems); 2) diabetes; 3) heart conditions; 4) hypertension; 5) chronic obstructive pulmonary disease (COPD) or asthma; 6) hearing impairments; 7) consequences of stroke; 8) cancer; 9) dysfunction of the thyroid gland;10) gastrointestinal conditions; 11) chronic allergies; 12) chronic skin problems; and 13) psychological problems. At baseline, only 9 of these 13 self-reported condition categories were entered in the prediction model because 4 of the condition categories were scarcely reported (i.e. chronic allergies, dysfunction of the thyroid gland, chronic skin and psychological problems) [10].

Furthermore, age and gender were taken from the patients' hospital charts. Distance visual acuity was assessed for all participants by their ophthalmologist. This was assessed by projection and with habitual correction for both eyes separately. To enable meaningful computations, decimal visual acuity values were transformed to logMAR values (-log_10_Visual Acuity), where higher values represent more vision loss, i.e., lower visual acuity values. According to the World Health Organization, low vision is defined as a visual acuity < 0.3 (logMAR ≥ 0.52) and/or a visual field < 20°, and blindness as a visual acuity < 0.05 (logMAR ≥ 1.30) and/or a visual field < 10°. Living independently (versus nursing home resident) and social status (married or single) were assessed by self-report. Rehabilitation type was either the optometric service or the multidisciplinary service, depending on the place of recruitment of the patient [15].

### Statistical analysis

#### Non-response, loss to follow-up and patient characteristics

Non-response from eligible patients at baseline, and between baseline and five-month follow-up, was calculated. To examine differences between participants who were still in the study after five months and those lost to follow-up, independent samples t-tests (EQ-5D_index_, number of co-existing conditions, age), χ^2^-tests (type of co-existing conditions, gender, independent living, social status, rehabilitation type) and Mann-Whitney tests (logMAR visual acuity) were used.

In addition to prevalence, the specific co-existing conditions were further explored by establishing which conditions reported at baseline were lost to follow-up, whether conditions reported at baseline were still reported at follow-up and, finally, which conditions were newly reported at follow-up. This information was reported to gain insight into the course of co-morbidity reports between baseline and follow-up. However, only baseline co-morbidity reports were entered into the regression models to assess the risk profile.

To investigate change in the number of self-reported co-existing conditions and logMAR visual acuity between baseline and follow-up, we used paired samples t-tests.

#### Health-related QOL

Before analyzing the prediction models, we started with some general analyses on the EQ-5D. To put the EQ-5D scores from the visually impaired older population into perspective, we compared baseline data of the visually impaired older patients (mean age 78 years) who reported having moderate or severe problems on the EQ-5D dimensions, with a visually impaired adult population (mean age 42 years) [11] and with an older group (aged 70–79 years) from the general population [21].

Using an independent samples t-test, we examined the difference in EQ-5D_index _between patients who reported to have co-morbidity and those who did not. To investigate overall change in QOL with the EQ-5D_index _between baseline and follow-up, we used a paired samples t-test.

#### Prediction model

To determine which self-reported co-existing conditions and patient characteristics predicted change in QOL after five months, linear regression analysis was used. Coefficients that were not significant (p > 0.05) were eliminated using a manual backward stepwise procedure. Change was defined by adjusting for the baseline scores of the EQ-5D_index _[22]; in this way, regression to the mean was corrected simultaneously. A consequence of regression to the mean is that, by chance, a change between baseline and follow-up is related to the initial value [22]. To compensate for missing values, sensitivity analyses for different assumptions were conducted by repeating the final prediction models. The sensitivity analyses provided similar results to those of the initial analyses and are therefore not reported here. To gain more insight into the prediction model, we also analyzed the data without correcting for the EQ-5D_index _baseline score; these analyses show which independent variables predict EQ-5D_index _scores after five-month follow-up. Beforehand, the linear assumption was assessed in univariate regression models and was considered satisfactory. In addition, co-linearity between variables was investigated. Pearson's correlations were highest between age and living in a nursing home (*r *= 0.29); all other correlations were (by far) lower than 0.3. Furthermore, residual and diagnostic analyses were checked for violation of the assumptions underlying the regression analyses. The distribution of residuals was considered normal. Data were analyzed using SPSS 14 for Windows.

## Results

### Non-response and loss to follow-up

A total of 357 patients were eligible for inclusion in the study; of these, 61 (17.1%) did not participate [13,15]. Of the remaining 296 patients who completed the baseline measurements, 50 (16.9%) were lost to follow-up after five months, and an additional 6 patients (2%) did not complete the five-month measurement of the EQ-5D. Of the 50 non-respondents, 10 patients died (3.4%), 35 (11.8%) were either unable to or no longer wished to participate, and 5 patients (1.7%) were either untraceable or the reason for non-response was unknown. Patients who were lost to follow-up after five months initially reported worse baseline EQ-5D_index _scores (mean 0.57; SD 0.29) than those who continued to participate in the study (mean 0.69; SD 0.24; p = 0.01). There were no major differences between the characteristics of the respondents at baseline and those of the non-respondents at five-month follow-up (Table 1). Similarly, there were no significant differences in the baseline reports of co-existing conditions between respondents and non-respondents at five-month follow-up (data not shown).

**Table 1:** Characteristics of the respondents at baseline, compared with those of non-respondents at five-month follow-up

**Patient characteristics**	**Respondents (n = 246)**	**Non-respondents (n = 50)**
Age in years: mean (SD)	78.0 (8.9)	80.3 (7.8)
Gender: female	62.6%	58.0%
LogMAR visual acuity best eye: median [IQR]	0.55 [0.42;0.77]	0.52 [0.41;0.80]
Social status: living alone	52.4%	62.0%
Independent living:	85.8%	78.0%
Rehabilitation type: optometric service	53.7%	58.0%
Co-morbidity:	75.5%	70.0%
		
Primary cause of visual impairment*		
Age-related macular degeneration	53.1%	50.0%
Diabetic retinopathy	13.2%	14.0%
Glaucoma	5.8%	8.0%
Cataract	5.3%	6.0%
Occluded vein	5.3%	6.0%
Other	17.3%	16.0%

SD = standard deviation; IQR = interquartile range;

*Cause of visual impairment in the eye with the better visual acuity as assessed at baseline during a routine clinical

eye examination by the participant's ophthalmologist.

### Patient characteristics

Table 1 presents the baseline characteristics of the patients. Of the visually impaired population, in more than 50% the primary cause of vision loss was AMD. Three patients (9.1%) who reported to suffer from the consequences of stroke (n = 33, Table 2) had suffered a cerebrovascular accident as the primary diagnosis of vision loss; 38 patients (52.1%) who reported diabetes (n = 73) had diabetic retinopathy as the primary cause of vision loss.

**Table 2:** Prevalence of co-existing conditions at baseline and the course of response during five months of follow-up.

**Co-existing conditions** **(n = 296)**	**Prevalence at baseline** **n (% of 296)**		**Not reported at follow-up** n (%*)		**Lost to follow-up** n (%*)		**New report at follow-up** **n (% of 246)**	
Diabetes	73	(24.7)	6	(8.2)	10	(13.7)	3	(1.2)
COPD or asthma	32	(10.8)	9	(28.1)	4	(12.5)	5	(2.0)
Heart	67	(22.7)	13	(19.4)	8	(11.9)	8	(3.3)
Stroke	33	(11.2)	6	(18.2)	5	(15.2)	8	(3.3)
Hearing impairments	23	(7.8)	10	(43.5)	5	(21.7)	9	(3.7)
Musculoskeletal	82	(27.8)	18	(22.0)	15	(18.3)	16	(6.5)
Cancer	13	(4.4)	2	(15.4)	4	(30.8)	3	(1.2)
Hypertension	48	(16.3)	14	(29.2)	11	(22.9)	12	(4.9)
Gastrointestinal	15	(5.1)	4	(26.7)	3	(20.0)	6	(2.4)

*Percentages calculated from the number of co-existing disease at baseline, e.g. 6/73 = 8.2%.

There was no significant change in LogMAR visual acuity between baseline (mean 0.66; SD 0.38) and follow-up (0.68; SD 0.40; p = 0.19), or in the mean number of co-existing conditions (mean 1.34; SD 1.0 versus mean 1.28; SD 1.0; p = 0.20).

Patients reported a median number of co-existing conditions of 1 (range 0–4), and 25% of the patients reported not to suffer from any co-existing conditions [10]. Table 2 shows that about 25% of the visually impaired population reported to have diabetes, heart conditions or musculoskeletal conditions, and that some patients did not report the co-existing conditions five months later or were lost to follow-up. For example, 43.5% no longer reported their hearing impairment, and 21.7% of patients who reported a hearing impairment at baseline were lost to follow-up. Moreover, 9 other patients 'newly' reported to have a hearing impairment at five-month follow-up.

### Health-related QOL

Table 3 presents the most common descriptive health states of the visually impaired older patients. The health state "21221" was reported by almost 12%; this indicates that these patients had moderate problems with 'mobility', no problems with 'self-care', moderate problems with 'daily activities' and 'pain or discomfort', and no problems related to 'anxiety or depression'. Furthermore, 40.4% reported to have health states other than those presented in Table 3; of those patients, 73 (24.7%) reported a health state with at least one '3', representing severe problems on one or more EQ-5D dimensions.

**Table 3:** Most frequently reported EQ-5D health states by the patients at baseline

**Health state**					**EQ-5D_index_**	**Patients**	
**MOB**	**SC**	**UA**	**PD**	**AD**		**n**	**%**
2	1	2	2	1	0.78	34	11.5%
1	1	1	1	1	1.00	28	9.5%
2	1	2	2	2	0.65	24	8.1%
2	2	2	2	1	0.69	15	5.1%
1	1	2	1	2	0.77	14	4.7%
2	1	2	1	1	0.86	14	4.7%
1	1	2	1	1	0.90	12	4.1%
2	1	1	1	1	0.89	12	4.1%
2	2	2	2	2	0.57	12	4.1%
2	1	1	2	1	0.81	11	3.7%
Other health profiles					--	120	40.4%

MOB (mobility); SC (self-care); UA (usual activities); PD (pain/discomfort);

AD (anxiety/depression); 1 (no problems); 2 (moderate problems); 3 (severe problems)

**Figure 1:**
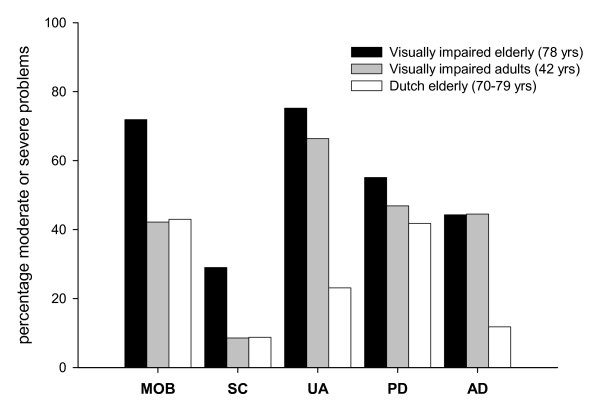
**Patients (%) reporting moderate or severe problems on EQ-5D-dimensions compared with the two reference groups**. MOB (mobility); SC (self-care); UA (usual activities); PD (pain/discomfort); AD (anxiety/depression).

Figure 1 presents baseline percentages of our visually impaired patients (mean age 78 years) who reported having moderate or severe problems on the EQ-5D dimensions. Those proportions were compared with a visually impaired adult population (mean age 42 years) [11], and with a general older population (aged 70–79 years) [21]. About 75% of our visually impaired older patient group reported moderate or severe problems on the 'usual activities' (moderate 55.9%; severe 19.3%) and 'mobility' dimensions (moderate 70.9%; severe 1.0%), followed by 'pain and discomfort' (moderate 48.0%; severe 7.1%), 'anxiety or depression' (moderate 39.2%; severe 5.1%) and 'self-care' (moderate 25.3%; severe 3.7%). This means that more of the visually impaired older patients reported having some or severe problems on all dimensions of the EQ-5D compared with both reference groups. However, the proportion of visually impaired older patients reporting problems related to 'anxiety or depression' was comparable to that reported in the reference group of visually impaired adults (44.5%). Nevertheless, a relatively larger group of visually impaired older patients reported having moderate or severe problems related to 'anxiety or depression' than older persons in the general population (11.8%).

**Figure 2:**
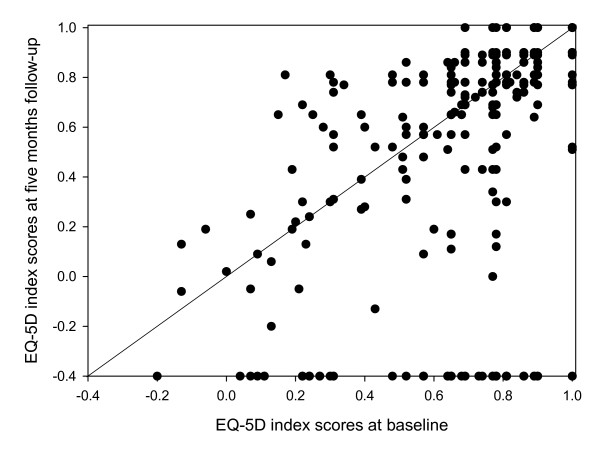
**EQ-5D_index _scores at baseline compared with those at five-month follow-up**. Solid line is the identity line; dots on the X-axis (at -0.4) represent baseline EQ-5D_index _scores of the patients lost to follow-up.

Figure 2 shows plots of the EQ-5D_index _scores at baseline and at five-month follow-up. A paired samples t-test showed that there was no significant change in EQ-5D_index _scores between baseline and follow-up. Furthermore, patients who reported to have co-morbidity, had significantly lower EQ-5D_index _baseline scores (mean 0.63; SD 0.26) than those who reported not to have co-morbidity (mean 0.76; SD 0.21; p < 0.001).

### Risk profile

The results of the regression analyses are presented in Table 4. It can be seen from the first column (all variables) that patients who reported diabetes, COPD/asthma, consequences of stroke, musculoskeletal conditions, cancer, gastrointestinal conditions or higher logMAR Visual Acuity values, experienced a lower QOL after five months compared to patients who did not report those conditions or who had lower logMAR values. In addition, it can be seen from the second column that in patients reporting COPD/asthma, consequences of stroke, musculoskeletal conditions or a higher LogMAR Visual Acuity, QOL declined during follow-up. Patients reporting diabetes, cancer or gastrointestinal conditions had no significant decline in QOL during the follow-up period. COPD/asthma, consequences of stroke, musculoskeletal conditions or a higher LogMAR Visual Acuity remained in the final prediction model after eliminating non-significant variables (p > 0.05); this means that those variables predicted a significant decline in EQ-5D_index _scores. These relevant prognostic variables explained 19.8% of the variance.

**Table 4:** Multivariate regression models for change in QOL between baseline and five-month follow-up

	**All variables**	**p-value**	**All variables adjusted for baseline**	**p-value**	**Relevant variables adjusted for baseline**	**p-value**
	β (SE)		β (SE)		β (SE)	
Baseline EQ5D score	--	--	0.62 (0.06)	<0.01	0.65 (0.06)	0.00
						
Diabetes	-0.07 (0.04)	0.05	-0.02 (0.03)	0.49	--	--
COPD or asthma	-0.12 (0.05)	0.01	-0.08 (0.04)	0.04	-0.09 (0.04)	0.02
Heart	-0.01 (0.04)	0.86	-0.02 (0.03)	0.51	--	--
Stroke	-0.16 (0.05)	<0.01	-0.10 (0.04)	0.02	-0.10 (0.04)	0.02
Hearing impairment	-0.09 (0.06)	0.12	-0.03 (0.05)	0.57	--	--
Musculoskeletal	-0.20 (0.04)	<0.01	-0.09 (0.03)	<0.01	-0.09 (0.03)	0.00
Cancer	-0.18 (0.08)	0.03	-0.06 (0.07)	0.37	--	--
Hypertension	0.04 (0.04)	0.35	0.02 (0.04)	0.60	--	--
Gastrointestinal	-0.17 (0.07)	0.02	-0.08 (0.06)	0.20	--	--
						
Age	0.00 (0.002)	0.73	0.00 (0.002)	0.74	--	--
Gender (female)	-0.05 (0.03)	0.14	-0.04 (0.03)	0.13	--	--
LogMAR VA	-0.14 (0.04)	<0.01	-0.09 (0.03)	0.01	-0.07 (0.03)	0.03
Social status (living alone)	-0.03 (0.03)	0.35	0.02 (0.03)	0.49	--	--
Independent living (nursing home)	-0.07 (0.05)	0.13	-0.03 (0.04)	0.43	--	--
Rehabilitation type (optometric service)	0.02 (0.03)	0.58	-0.02 (0.03)	0.49	--	--

β:unstandardized regression coefficient; SE: standard error

## Discussion

Our study aimed to provide a risk profile for visually impaired older patients related to a change in QOL. First, when not taking specific risk factors into account, for the entire group there was no significant change in health-related QOL between baseline and five-month follow-up, as measured with the EQ-5D_index_. However, we expected this result to be an underestimation of the decline in QOL because patients with worse scores were lost to follow-up. With the risk profile presented in this study it was possible to determine patients at risk for a relatively rapid decline in QOL, in addition to patients who already experienced a low QOL. Patients who reported at baseline to have diabetes, COPD/asthma, consequences of stroke, musculoskeletal conditions, cancer, gastrointestinal conditions or higher logMAR Visual Acuity values (which means more vision loss) experienced a lower QOL after five months compared to patients who did not report those conditions or who had lower logMAR Visual Acuity values. Patients reporting those conditions (besides their eye condition) or patients with more vision loss can be considered target groups who need more attention. Ophthalmologists may consider referral to another sub-specialty if the patient is currently not under treatment for the condition(s) that they have reported. A referral by the ophthalmologist or optometrist to a multidisciplinary rehabilitation service seems appropriate for patients with multiple conditions. In addition to reading aids, these patients may need occupational therapy, specialized mobility training, more extensive training for using low-vision aids or help from a social worker, to adapt to their visual disability. Furthermore, in visually impaired older patients we found that having COPD/asthma, consequences of stroke, musculoskeletal conditions or more vision loss predicted a relatively rapid decline in QOL between baseline and five-month follow-up. The fact that patients with diabetes, cancer and gastrointestinal conditions did not show a further decline in QOL might indicate that they were under treatment by a clinician or general practitioner during the study period.

Our results concur with those of Sprangers et al., who explored the relative impact of diseases on QOL in a large group of patients with a wide range of chronic conditions [23]. They reported that patients with gastrointestinal, cerebrovascular and musculoskeletal conditions experienced the most detrimental impact, those with visual impairments and chronic respiratory conditions experienced an intermediate impact and, for example, hearing impairments or dermatological conditions appeared to result in a relatively favorable impact [23].

The results of our study showed that visually impaired older patients frequently suffer from one or more co-existing conditions (other than their eye condition), and that these patients experienced a lower health-related QOL than patients without any self-reported conditions at baseline. However, it has been reported that clinicians find it difficult to appreciate the impact of low vision on QOL [24]. Therefore, it might be helpful for ophthalmologists to understand that low vision and those specific co-existing conditions cause a measurable extra burden or even a rapid decline in QOL in older patients. These older patients already experience a worse QOL than, for example, younger visually impaired patients; this was shown by comparison with reference populations among visually impaired adults [11], and older adults in the general Dutch population [21]. In contrast, the fact that our visually impaired older patients were referred to rehabilitation services by their ophthalmologist (e.g. to an optometrist or to a multidisciplinary service) demonstrates that the ophthalmologist was at least aware of the disabling problems caused by the low vision of their patients. Although a referral did not necessarily increase the patient's health-related QOL (which is not expected from low-vision rehabilitation services), an improvement was observed in some of the vision-related QOL domains. In a previous non-randomized study among the same group of visually impaired older patients, we used a disease-specific questionnaire to measure the effect of low vision rehabilitation in terms of vision-related QOL [13]. These latter patients showed an improvement on the 'reading small print' dimension after five months, for both rehabilitation types (optometrist/multidisciplinary service). Patients who went to the multidisciplinary center also improved on the 'adjustment' to vision loss dimension after five months. Both dimensions were part of the Low Vision Quality of Life questionnaire [25]. On this questionnaire, the 'basic aspects' of vision, vision-related 'mobility' and 'visual (motor) skills' dimensions did not change significantly after five months. In general, rehabilitation for patients with irreversible eye conditions is recommended. For example, in the case of AMD there is usually no medical treatment available so that rehabilitation is the only option to adjust to living with a visual disability.

Our study has some limitations. Co-morbidity was assessed with an open-ended question, and this questioning method can result in under-reporting compared to more specific methods [26]. Open-ended questions are considered sub-optimal for assessing the prevalence of co-existing conditions, because in that case mainly the serious conditions are reported [27]. In our study it is feasible that the visually impaired older patients reported those conditions that had the most impact on their QOL at the time of the measurements. Moreover, when we investigated the reliability of the self-reported conditions we observed that between baseline and follow-up the reports on co-morbidity were not stable. One reason for this was loss to follow-up, and the other was that the patients did not continue to report the co-existing conditions which they had reported at baseline. Moreover, some patients reported co-existing conditions for the first time at the follow-up measurement. It is not clear whether these changes in self-reports reflect a true change, or simply a lack of reports at baseline for which the reasons are not clear. It is possible that patients were not aware of their condition at both of the measurement points, either because the symptoms were absent or because they had problems with recollection. Alternatively, at follow-up the patients might have thought that the researchers were already aware of their co-existing conditions because they had reported them at baseline; in this case they might have considered it superfluous to report their (chronic) co-existing condition(s) a second time. In contrast, Klabunde et al. showed that patients were generally able to provide reliable reports of their co-existing conditions over time; however, arthritis had the highest proportion of inconsistent responses [28]. More insight into the validity of self-reported co-morbidity in open-ended questions was revealed from our previous study. In that study, for most condition categories there was a lack of agreement between co-morbidity reports of patients and those of their GP; the agreement differed per condition, where patients mostly under-reported. However, for diabetes, COPD/asthma and heart conditions we found very good to moderate agreement between the patients and the GPs [10].

The current study did not include a thorough investigation of the nature of open-ended questions. More research is needed to establish the reliability of open versus closed-ended questions administered by patients. Pre-structured questionnaires are available [29], which should provide a more complete view of the patient's co-morbidity than open-ended questions [27]; these are easier to complete by older patients because they depend less on the recollection ability of the patients. We do note, however, that open-ended questions give a more accurate reflection of how co-morbidity is usually addressed in a clinical setting [26]. Although we do not have exact information concerning the patient's co-morbidity, in the clinic one is also confronted with the incompleteness of patient reports. Nevertheless, we found that self-reported co-morbidity from open-ended questions predicted a decline in QOL, with results comparable to those of larger studies [23].

Finally, the EQ-5D is one of the most widely used generic index measures of health-related QOL [30] and is increasingly used as a stand-alone measure [31]. The questionnaire allowed us to gain insight into various health states, to compare different sub-groups of our patient population, and to compare our study population with two reference groups. However, the EQ-5D has been criticized for having only three response categories per dimension, which could lead to lack of measurement precision and responsiveness (see e.g. Pickard et al.) [32]. For example, on the mobility dimension it seems to be a large step for patients to choose between the response categories 2) and 3): where 1) represents no problems with walking about, 2) moderate problems with walking about, and 3) being confined to bed. Therefore, the results of our study on QOL decline may even be an underestimation of the actual QOL decline in visually impaired older patients. Furthermore, in the field of ophthalmology and low vision it is increasingly more common to use Rasch analysis or other item response theory models to calculate health-related outcome measures, such as QOL questionnaires [13,33,34]. Some efforts have been made to use Rasch analyses on the five dimensions to validate the EQ-5D [32]; however, problems still exist with these valuations and they have not yet been widely accepted. For comparability purposes it has been recommended to follow the original validated and widely used valuations [30].

## Conclusion

We believe that the knowledge of specific co-existing conditions is important for public health, the patient's individual care and the ophthalmologist whose patients consist mainly of older adults. Patient's self-reported co-morbidity and other characteristics may influence the ophthalmologist's medical decision-making concerning surgery, or their approach to older patients who often have complicated drug regimens [35]. Although our results should be confirmed in an additional study with pre-structured co-morbidity questionnaires, this study shows that visually impaired older patients with specific co-existing conditions and low vision experienced a lower QOL at follow-up or were at higher risk of a rapid decline in QOL.

In conclusion, we recommend to actively ask visually impaired older patients about their musculoskeletal conditions, COPD/asthma and consequences of stroke, and to continue referring patients with low vision to rehabilitation services, according to the guidelines developed in the USA [36] and in the Netherlands [9]. With a risk profile, as presented in this study, a rehabilitation intervention or a specific referral to another sub-specialty may be of benefit for the health and vision-related QOL of the patient and for the involvement of ophthalmologists in their patient's general health.

## Abbreviations

AMD: Age-related macular degeneration; COPD: Chronic obstructive pulmonary disease; EQ-5D: EuroQol 5-Dimensions; EQ-VAS: EuroQol Visual Analogue Scale; GP: General practitioner; LogMAR VA: Logarithm of the Minimum Angle of Resolution – Visual Acuity; QOL: Quality of life

## Competing interests

The authors declare that they have no competing interests.

## Authors' contributions

RMAVN drafted the manuscript and performed all statistical analyses; MRDB participated in the design of the study, collected data, advised on the statistical analyses, and helped to interpret the data; JGJH drafted a preliminary version of the manuscript and performed data analyses; PJR helped to draft the manuscript and revised the manuscript for important intellectual content; GHMBVR conceived of the study and its design; helped to draft the manuscript, and has given final approval of the version to be published; All authors read and approved the final manuscript.
